# Orientation of Electrospun Magnetic Nanofibers Near Conductive Areas

**DOI:** 10.3390/ma13010047

**Published:** 2019-12-20

**Authors:** Jan Lukas Storck, Timo Grothe, Al Mamun, Lilia Sabantina, Michaela Klöcker, Tomasz Blachowicz, Andrea Ehrmann

**Affiliations:** 1 Bielefeld University of Applied Sciences, Faculty of Engineering and Mathematics, 33619 Bielefeld, Germany; jan_lukas.storck@fh-bielefeld.de (J.L.S.); Timo.grothe@fh-bielefeld.de (T.G.); Al.mamun@fh-bielefeld.de (A.M.); Lilia.sabantina@fh-bielefeld.de (L.S.); Michaela.kloecker@fh-bielefeld.de (M.K.); 2 Silesian University of Technology, Institute of Physics—CSE, 44-100 Gliwice, Poland; tomasz.blachowicz@polsl.pl

**Keywords:** electrospinning, magnetic nanofibers, magnetite, magnetic field lines, Ampère’s right-hand grip rule, Maxwell equations

## Abstract

Electrospinning can be used to create nanofibers from diverse polymers in which also other materials can be embedded. Inclusion of magnetic nanoparticles, for example, results in preparation of magnetic nanofibers which are usually isotropically distributed on the substrate. One method to create a preferred direction is using a spinning cylinder as the substrate, which is not always possible, especially in commercial electrospinning machines. Here, another simple technique to partly align magnetic nanofibers is investigated. Since electrospinning works in a strong electric field and the fibers thus carry charges when landing on the substrate, using partly conductive substrates leads to a current flow through the conductive parts of the substrate which, according to Ampère’s right-hand grip rule, creates a magnetic field around it. We observed that this magnetic field, on the other hand, can partly align magnetic nanofibers perpendicular to the borders of the current flow conductor. We report on the first observations of electrospinning magnetic nanofibers on partly conductive substrates with some of the conductive areas additionally being grounded, resulting in partly oriented magnetic nanofibers.

## 1. Introduction

Nanofibers can be produced by electrospinning diverse polymers or polymer blends [1,2,3]. Generally, different technologies can be applied, such as the common needle-based one in which a polymer solution or melt is pressed through a needle into the electric field [4,5,6], wire-based techniques [7,8,9], cylinder-based one [10] and diverse other geometries of spinning electrode and substrate. Such nanofiber mats can be used for a broad variety of applications, such as air or water filtration [11,12,13], biotechnology and biomedicine [14,15,16,17], batteries and solar cells [18,19,20], etc.

Typically, such nanofiber mats contain arbitrarily oriented nanofibers. For several applications, however, aligning them would be supportive. In the literature, different approaches can be found to reach this goal. Often a high-speed rotating collector is used [21,22,23], but fiber orientation can also be reached by using a pair of blades as the collector [24,25] or stretching the electrospun mat in ethanol [2].

Especially for the case of magnetic nanofibers, a magnetic field-assisted electrospinning process was suggested to create oriented nanofibers [26,27,28]. A high alignment is of great interest for magnetic nanofibers to create a high magnetic anisotropy, based on the shape anisotropy of such fine nanofibers [29,30,31]. Aligned electrospun magnetic nanofibers may also close the gap between fully determined nanofibers, as used in the racetrack memory [32], and arbitrarily oriented magnetic nanofibers [33], to enable stochastic behavior in biologically-inspired neuromorphic computing [34]. On the other hand, such aligned magnetic nanofibers were shown to have high longitudinal elasto-magnetic strain and transverse magnetic deflection, making them useful for microelectronic or biomedical applications in which deformations occur or a magnetic anisotropy is necessary [26]. They can be used in situ as scaffolds that topographically guide cell growth, e.g., of neurites [35] or mesenchymal stem cells [36] growing significantly farther and better aligned on such aligned fibers than on a pure hydrogel. On the other hand, tensile strength and thermal conductivity of such nanofiber mats or corresponding nano-composites were found to be significantly enhanced due to fiber alignment [37]. Aligned magneto-mechanically spun nanofibers showed a high stretchability and a fast and reproducible pressure response, making them well suited as pressure sensors in stretchable devices [38].

Adding a magnetic field or working with a rotating cylinder, however, is often not possible especially in commercial electrospinning devices with usually high safety standards, impeding modifications of the interior. Here we report for the first time on another possibility to align magnetic nanofibers during electrospinning which can easily be applied in many electrospinning devices. Due to the strong electric field in the spinning chamber, conductive lines applied on an isolating substrate lead to a fiber orientation perpendicular to the borders between conductive and isolating areas. We report on the influence of grounding the conductive lines, spinning duration and other spinning parameters.

## 2. Materials and Methods

**Figure 1:**
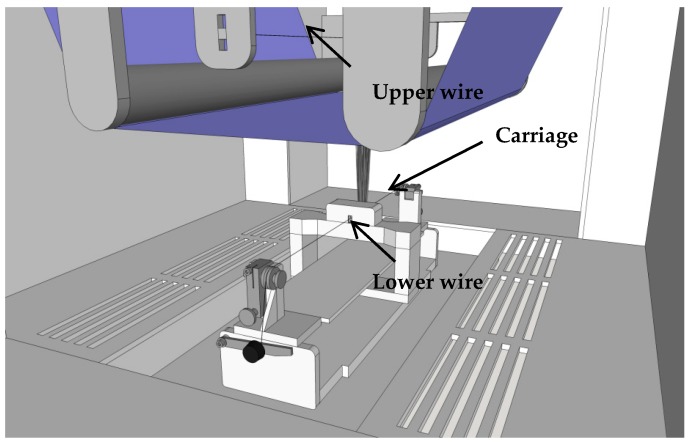
Sketch of the Nanospider Lab. From [33].

Nanofiber mats were created by electrospinning in a “Nanospider Lab” (Elmarco Ltd., Liberec, Czech Republic), using a wire-based technology. The setup is shown in Figure 1. The carriage contains the spinning solution which flows through a spinning nozzle. In this way the lower wire is coated with polymer solution. Since a large positive voltage is applied to the lower wire, while the upper wire is grounded, a strong electric field is created in the spinning area which is usually strongest in the middle, between the two wires. Along the edges of the blue-marked substrate, the electric field becomes non-uniform, resulting often in partly aligned nanofibers which can be recognized if the substrate is not moved during the experiments, as done here.

It should be mentioned that this electrospinning technology is not well-suited to prepare very long nanofibers in the range of several millimeters, as it is possible with the needle-based process [39,40,41]. Due to the usually isotropic arrangement and the interconnections formed between the fibers, length estimation of fibers spun by a wire-based technology is difficult and, to the best of our knowledge, not yet reported in the literature.

The spinning parameters for this study were: voltage between the wires 80 kV (with a positive voltage applied to the spinning wire and the other wire behind the substrate being grounded), nozzle diameter 0.9 mm (pure polyacrylonitrile nanofiber mats) or 1.5 mm (magnetic nanofiber mats), carriage speed 100 mm/s, substrate speed 0 mm/s, bottom electrode/substrate distance 240 mm, ground electrode / substrate distance 50 mm, temperature 24–26 °C and relative humidity in the chamber 31%–32%. Spinning was carried out for 10 min if not mentioned differently.

For the polymer solution, 16% polyacrylonitrile (PAN) (X-PAN, Dralon, Dormagen, Germany) were dissolved in dimethyl sulfoxide (DMSO) (min 99.9%, S3 chemicals, Bad Oeynhausen, Germany) and stirred for 2 h at room temperature on a magnetic stirrer. Magnetic Fe_3_O_4_ (magnetite) nanoparticles with particle sizes of 50–100 nm (Merck KGaA, Darmstadt, Germany) were added by manually stirring the solution for 10 min before dispersion in an ultrasonic bath for 30 min at 35 °C with a frequency of 37 kHz. The polymer:nanoparticle weight ratio was chosen as 1:1.56 which is in the range of a former test series [33] and thus known to be well spinnable. Pure PAN nanofiber mats were electrospun under identical conditions as a reference.

To produce conductive areas on the polypropylene (PP) non-woven which is commonly used as a substrate in the Nanospider Lab, copper tape (width 5 mm, purchased from dooppa, Hubei, China) was glued on the PP substrate, either parallel or perpendicular to the electrospinning wires, and in some cases grounded by connecting one of their ends electrically with the grounded electrospinning wire.

The resulting nanofiber mats were investigated by a digital microscope VHX-600D (Keyence, Neu-Isenburg, Germany), a confocal laser-scanning microscope (CLSM) VK-8710 (Keyence), a scanning electron microscope (SEM) Zeiss 1450VPSE (Oberkochen, Germany) and a camera Canon 1300D with a lens Tamron SP AF 17–50 mm F/2.8 XR Di II LD Aspherical [IF]. Investigations of fiber diameters, angular distribution and fast Fourier transform (FFT) evaluations were performed with ImageJ 1.51j8 (National Institutes of Health, Bethesda, MD, USA).

## 3. Results and Discussion

**Figure 2:**
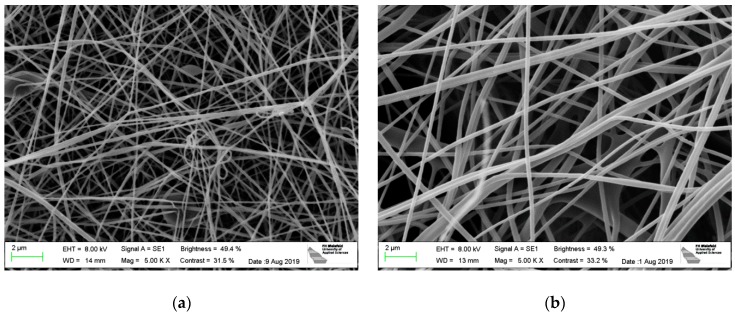

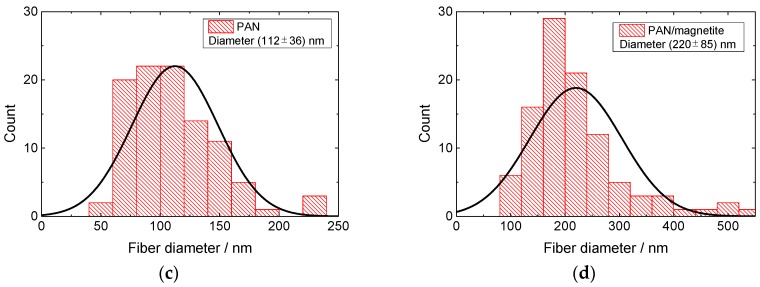
Scanning electron microscope (SEM) images of (**a**) pure polyacrylonitrile (PAN) nanofibers; (**b**) PAN/magnetite nanofibers; diameter distributions of (**c**) pure PAN nanofibers; (**d**) PAN/magnetite nanofibers. The x-axis of Figure 2c,d differ.

Firstly, Figure 2 shows a comparison of SEM images of electrospun nanofibers from pure PAN (Figure 2a,c) and from PAN/magnetite (Figure 2b,d). Both images are taken outside conductive areas to avoid possible effects due to modifications in the conductivity of the substrate.

As visible in Figure 2a,b, the magnetite nanoparticles result in a visible increase of the average fiber diameters. This finding is underlined by the diameter distributions given in Figure 2c,d for an overall number of 100 fibers each. Generally, both materials show smooth, straight fibers with relatively large bending radii, while especially in case of pure PAN several bifurcations are visible.

It should be mentioned that the finer fibers tend to be not separately visible in the CLSM image, resulting in apparently larger diameters of the nanofibers depicted in the latter.

**Figure 3:**
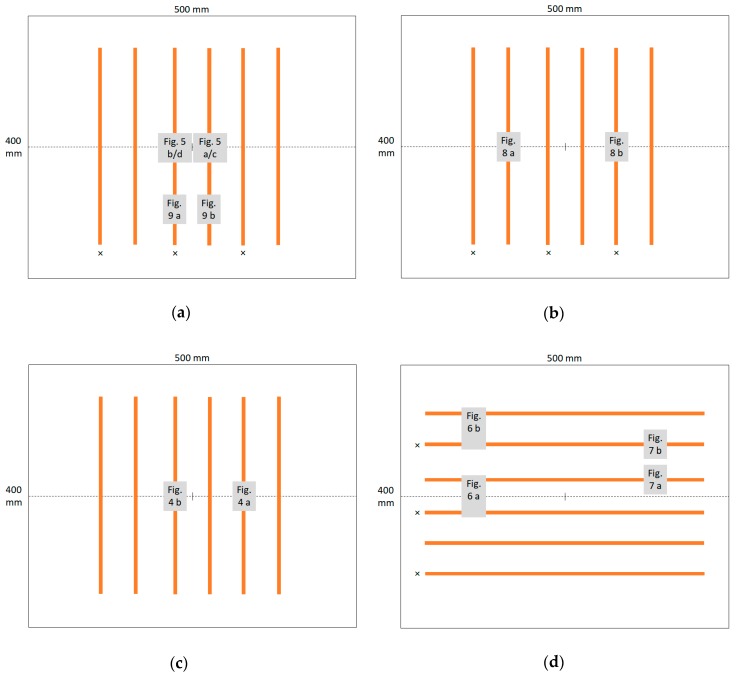
Overview of sample positions (x marks the grounded copper tapes, the dotted line marks the spinning wires): (**a**) PAN/magnetite spun for 10 min; (**b**) PAN/magnetite spun for 5 min; (**c**) pure PAN spun for 10 min; (**d**) PAN/magnetite spun for 10 min.

Next, Figure 3 gives an overview of the positions where the images depicted in this paper were taken.

The copper tapes (orange lines) are either aligned perpendicular to the spinning wires (dotted horizontal line) or parallel to it. Electrospinning was performed for 10 min or 5 min with PAN/magnetite or pure PAN.

These copper wires, especially when grounded, serve as additional ground electrodes, strongly influencing the electric field. Generally, simulations of the electric field in needleless electrospinning have revealed strong differences of the electric field near the connection line between both electrodes and farther away. Ahmad et al., e.g., showed that for a modified spinning wheel with teeth the highest electric fields were only available in a range of few cm and dropped fast outside this region [42]. Electrical field variations by approx. a factor of 7 were found by Wei et al., using an annular spinneret, along a few centimeters between the spinneret ring and its middle [43]. Wei et al. simulated similar electric field distributions for a metal dish spinneret [44]. Chen et al. as well as Niu et al. [45] compared another annular nozzle with the wire-based spinning principle and found higher charge densities at the very ends of the latter [46] where—in case of our wire-based electrospinning setup—no polymer solution is coated on the wire, while along the longest part of the wire, the field was uniform. It should be mentioned that in addition, even smallest variations of the distance between both wires can significantly influence the electric field strength [47]. The grounded copper tapes will, thus, not only modify the field distribution, but also the field strength.

While such simulations of the electric field distribution can be found in the literature for diverse nozzle and substrate geometries, similar theoretical investigations of the influence of the charge transfer from electrostatically charged nanofibers onto the ground electrode, or resulting magnetic fields or of moving magnetic fibers inside the electric field of the electrospinning setup are, to the best of our knowledge, not yet available. This paper thus serves as a base for future simulations of these dynamic procedures, pointing out phenomena occurring during electrospinning magnetic nanofibers and allowing for comparing future simulations with the experiment.

**Figure 4:**
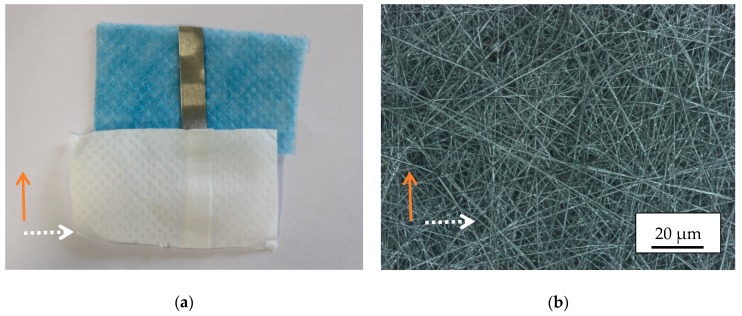
Non-magnetic reference nanofiber mat, electrospun partly on a conductive copper tape. (**a**) Photographic image; (**b**) confocal laser-scanning microscope (CLSM) image. The orange arrow marks the copper tape orientation, the white dotted arrow the spinning wire orientation.

Figure 4 depicts images of the nonmagnetic reference, electrospun on a non-grounded copper tape. As it is clearly visible in Figure 4a, the density of the nanofiber mat on the conductive area is slightly increased, as compared to the area outside. In the CLSM image (Figure 4b), no fiber orientation can be recognized along the border between conductive (top of the image) and non-conductive substrate (bottom). These findings remain unchanged for grounded copper tape and are independent from the orientation of the copper tape (perpendicular or parallel to the electrospinning wires).

**Figure 5:**
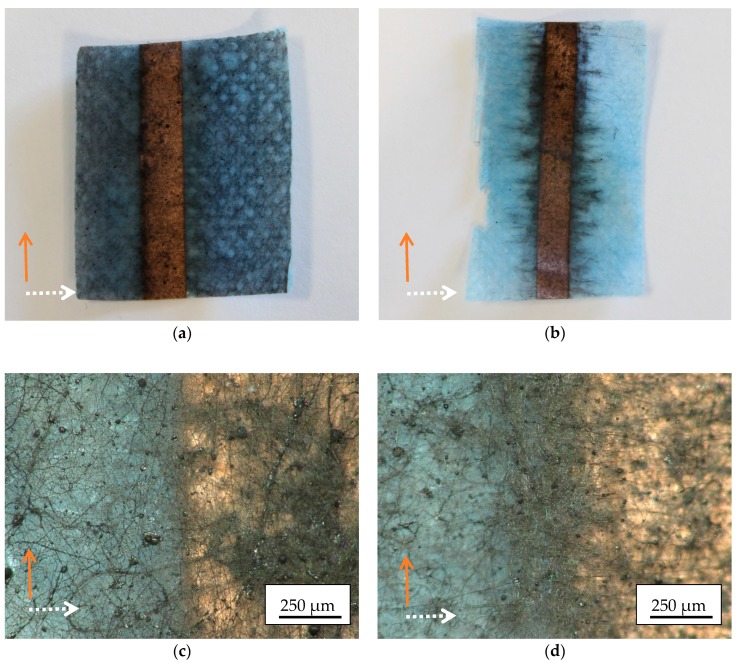
Images of magnetic nanofiber mats electrospun on copper tape which is oriented perpendicular to the electrospinning wires: photographic images on copper tape (**a**) not grounded; (**b**) grounded; microscopic images on copper tape; (**c**) not grounded; (**d**) grounded.

Next, Figure 5 shows photographic images of magnetic nanofiber mats, electrospun on grounded or not grounded copper tape, with the copper tape oriented perpendicular to the spinning wires.

The photographic images reveal a clear difference between the nanofiber mats electrospun on not grounded and grounded copper tape. On the latter, the nanofibers seem to be strongly concentrated along the borders of the copper tape, and from the macroscopic view, there is an orientation of the dark nanofibers perpendicular to the borders of the copper tape visible.

This finding, however, is not supported by the microscopic images (Figure 5c,d). While there is indeed a thicker nanofiber mat visible along the border of the grounded copper tape (Figure 5d), the nanofibers do not show a clear orientation on the microscale.

**Figure 6:**
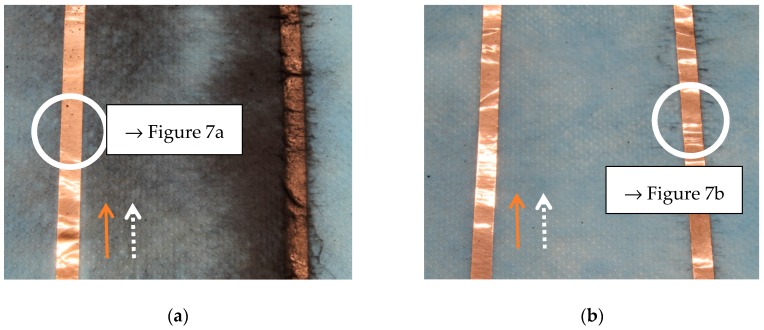
Photographic images of magnetic nanofiber mats electrospun on copper tape which is oriented parallel to the electrospinning wires: (**a**) in the middle of the substrate; (**b**) at the substrate border. In both images, the left strip is not grounded, while the right strip is grounded. The circles indicate the areas where the microscopic images in Figure 7a,b were taken.

Next, Figure 6 shows photographic images of the nanofiber mats, taken in the middle (Figure 6a) or at the border of the electrospinning area (Figure 6b), electrospun on not grounded (left strip in each image) or grounded copper tapes (right strip in each image) which are oriented parallel to the spinning wires. In Figure 6a, the grounded wire of the Nanospider Lab is positioned in the middle between the tapes visible here.

Firstly, it is clearly visible that many more fibers can be found on the grounded tapes than on the others. This could be expected since the electrospinning process works by creating a strong electric field between the high-voltage wire which is coated by the polymer solution and a grounded wire. Here, more grounded wires are created, in this way deforming the electric field and partly taking over this task from the original grounded wire of the machine.

Next, many more fibers are visible around the wires in the middle of the spinning area. Generally, this behavior is identical to the effect in the normal electrospinning process, usually resulting in a constant movement of the substrate if a large nanofiber mat with equal thickness along the whole area is desired. Here, however, this effect is much stronger since the grounded electrodes are now directly in the substrate plane, causing the electric field to vary laterally more strongly than in case of the normal grounded wire 50 mm above the substrate plane.

**Figure 7:**
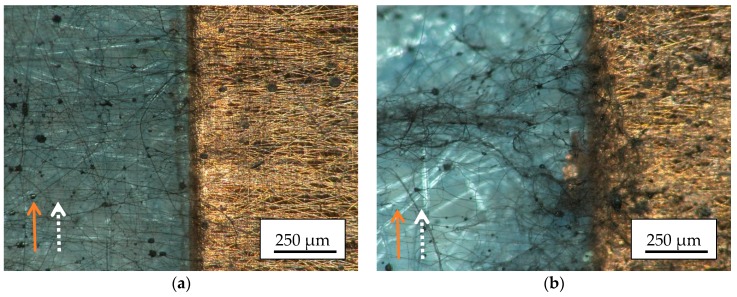
Microscopic images of magnetic nanofiber mats electrospun on copper tape which is oriented parallel to the electrospinning wires: (**a**) not grounded; (**b**) grounded.

Next, Figure 7 shows microscopic images of the nanofiber mats, electrospun on grounded or not-grounded copper tapes which are oriented parallel to the spinning wires.

Interestingly, especially in case of the not grounded copper tape (Figure 7a), there is a clear fiber orientation perpendicular to the copper tape, on top of it as well as in the direct environment. For the grounded tape (Figure 7b) on which again more nanofibers are accumulated although the image was taken near the border of the spinning area, there are also nanofibers visible which are oriented perpendicular to the border of the copper tape, but they are superposed by a bunch of nanofibers with nearly arbitrary orientation on top. Generally, Figure 7b shows fewer fibers than Figure 5b since the latter was taken near the middle of the spinning area.

This finding suggests that in the case of a copper strip aligned perpendicular to the electrospinning wires, the lack of alignment is also due to the large number of nanofibers. Fewer nanofibers deposited on the substrate, due to a shorter spinning time or a position close to the limits of the spinning range, can on the other hand lead to aligned nanofibers.

**Figure 8:**
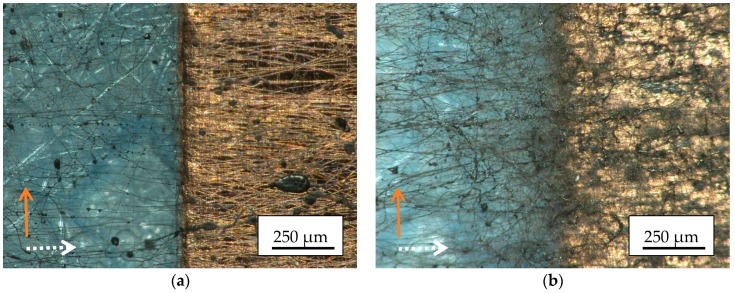
Microscopic images of magnetic nanofiber mats along the borders of the spinning area, electrospun for 5 min on copper tape which is oriented perpendicular to the electrospinning wires: (**a**) not grounded; (**b**) grounded.

The results of the next experiment with a reduced amount of nanofibers are presented in Figure 8. For this purpose, electrospinning was carried out for only 5 min (instead of 10 min before). Images taken along the borders of the spinning area reveal indeed a fiber orientation perpendicular to the copper tape, especially in case of the not grounded tape on which fewer nanofibers are positioned (Figure 8a).

Apparently, the orientation of the magnetic nanofibers with respect to the borders of the conductive areas is universal as long as not too many magnetic nanofibers are involved, independent from the orientation of the copper tape with respect to the spinning wires and from a possible grounding of the copper tape.

The electrospinning process, treated from the electromagnetic perspective, is a very complex and interesting phenomenon, not fully understood till today, and not yet deeply analyzed with the use of basic physical principles. For the technological case applied in the study reported here, we should distinguish between the two main components of the phenomenon; the movements of highly charged fibers before deposition on the substrate, and the discharging effects just after deposition. Moreover, these two situations can be further enhanced by the presence of electrically conductive copper tape, especially for the grounded case.

In the first phenomenon, the movement of electrons located in non-conducting fibers, in the moment before deposition, can be treated as electric currents flowing into directions perpendicular to the tape surface. In accordance to Ampère’s law, the current produces closed loops of magnetic field lines lying in-plane at the tape. Such a magnetic field enhances orientation order of ferromagnetic fibers which are deposited in a parallel order.

During the second step, when fibers are coupled physically to the tape, especially for the grounded case, the electrons flow from the fibers into the conducting materials, and discharging effects take place, in such a way that electrons from higher electrified fibers flow into places in where there are fewer electrons. Generally, this leads to a current flow along the conductive tapes and correspondingly to magnetic fields circularly surrounding the conductive tapes, i.e., perpendicular to them. In case of grounded copper tapes, the current flows towards the grounded end of the tape; in the case of the non-grounded tapes, only short-distance current flows occur so that the whole wire is homogenously charged. These effects of random local origin are a reason to spatially chaotic distribution of electrospun material, and obviously should dominate for the not-grounded tape, in where the random distribution of fibers is a result of spatially random electric-charge flows on the substrate. Hence, the parallelism of fibers, for the grounded case, is more evident for the case when a copper tape is perpendicular the electrospinning wires which define some electric field anisotropy.

**Figure 9:**
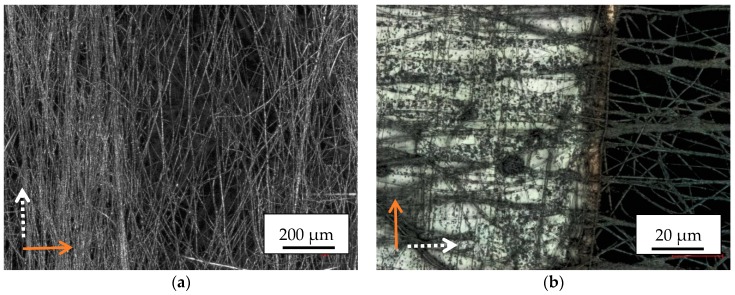
CLSM images of magnetic nanofiber mats, electrospun on copper tape which is oriented perpendicular to the electrospinning wires: (**a**) grounded, spun near the border (with the copper tape located below the lower image border oriented from left to right); (**b**) not grounded, in the middle of the sample. The magnifications differ (cf. inset scales). The orange arrows mark the copper tape orientations.

Figure 9 shows exemplary CLSM images in different magnifications, taken directly beside the border of the copper tape (Figure 9a, with the copper tape parallel to the lower border) or along the border, respectively. Again, the clear orientation of the nanofibers perpendicular to the conductive path is visible, while in both cases also nanofibers with different orientation can be found. It should be mentioned that in Figure 9b, the brown color along the edge of the copper strip indicates some agglomerations of pure magnetite nanoparticles in this area.

Generally, it must be mentioned that the alignment of the nanofibers perpendicular to the grounded copper tapes is much more efficient than the alignment perpendicular to the non-grounded ones. This is especially well visible in Figure 5a,b and Figure 6. These figures, however, also show why this alignment is lost for longer spinning durations in the middle of the spinning area: The positive charges flow from the flying nanofibers to the non-grounded copper tape and accumulate on it, thus blocking the identically charged fibers after several minutes of electrospinning, the grounded copper tapes work identically to the ground electrode, are steadily discharged and thus attract nanofibers continuously. This effect, which is normally desired to obtain a thick nanofiber mat, now destroys the alignment of the nanofibers impinging near the copper tape and the already aligned first “layer” of nanofibers since the newly arriving nanofibers cannot reach the copper tape surface, are thus not discharged as effectively as the first ones and in this way no longer create magnetic fields circularly surrounding the conductive tapes.

**Figure 10:**
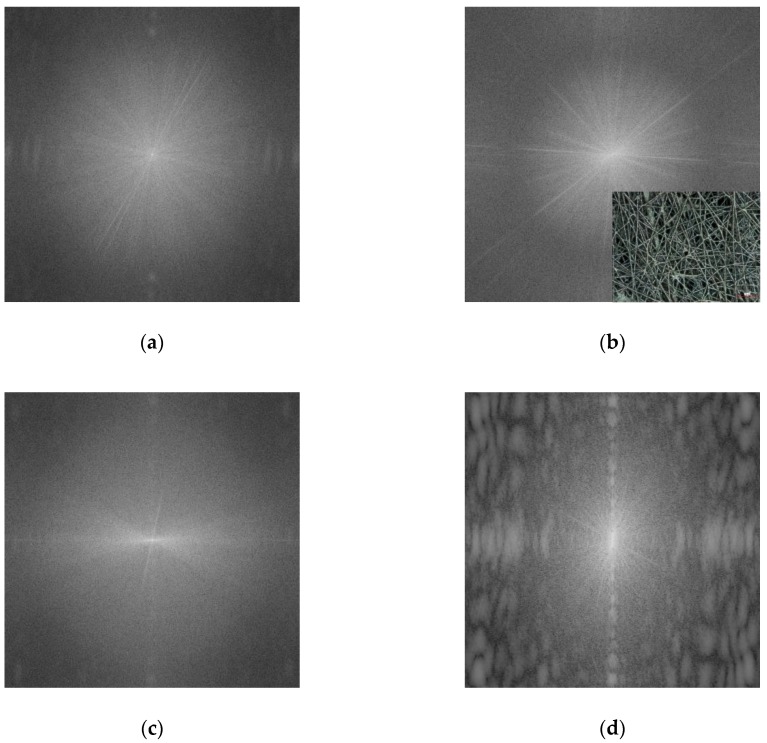
Fast Fourier transform (FFT) analyses of CLSM images, taken on (**a**) pure PAN on copper tape (Figure 4b); (**b**) PAN/magnetite nanofiber mat, spun on non-conductive substrate (cf. inset image); (**c**) PAN/magnetite nanofiber mat, spun on grounded copper tape (Figure 9a); (**d**) PAN/magnetite nanofiber mat, spun on not grounded copper tape (Figure 9b, right side of the image without copper visible only).

After these qualitative examinations, the fiber orientations are finally examined more quantitatively. For this, Figure 10 depicts a 2D FFT analysis of CLSM images, taken on PAN as well as PAN/magnetite nanofiber mats spun on different substrate positions. Generally, such FFT images can be expected to show a radially diffuse pattern for non-aligned fibers, while single lines in the FFT image hint at oriented fibers [48].

For the pure PAN nanofiber mat (Figure 10a) and the PAN/magnetite nanofiber mat, spun outside conductive areas (Figure 10b), no orientation is visible in the FFT images. This changed for the PAN/magnetite nanofiber mat, spun on grounded copper tape, as depicted in Figure 8a—here a possible fiber orientation becomes visible, suppressed by the relatively low contrast between neighboring fibers (Figure 9a), making the fiber orientation better visible by eye. In Figure 10d, however, a clear fiber orientation is visible from the FFT graph, underlining the fiber orientation which could already recognized qualitatively in Figure 9b.

**Figure 11:**
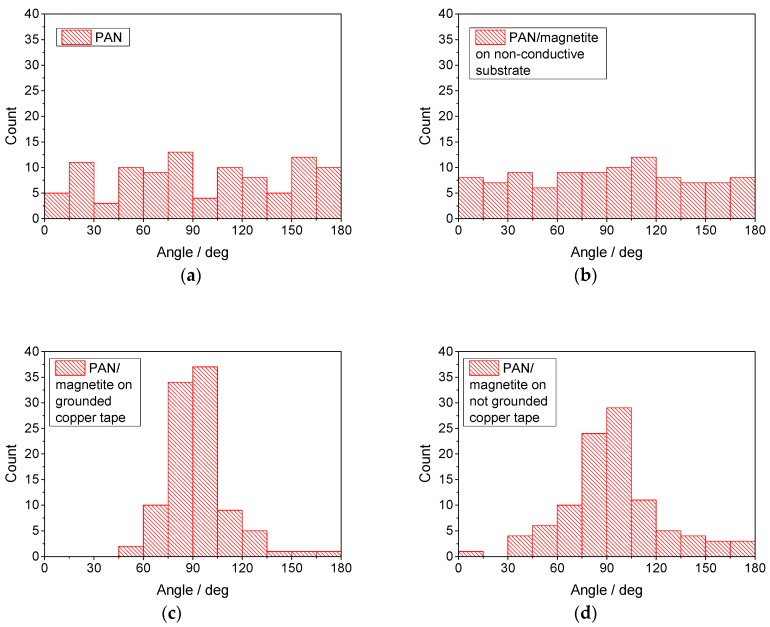
Fiber angle distributions derived from CLSM images, taken on (**a**) pure PAN on copper tape (Figure 4b); (**b**) PAN/magnetite nanofiber mat, spun on non-conductive substrate (cf. inset of Figure 10b); (**c**) PAN/magnetite nanofiber mat, spun on grounded copper tape (Figure 9a); (**d**) PAN/magnetite nanofiber mat, spun on not grounded copper tape (Figure 9b, right side of the image without copper visible only).

In addition, Figure 11 shows histograms of the fiber orientations with respect to the copper tape edges (if available) or with respect to the lower border of CLSM images, if the images were taken at other positions. The images chosen as bases are identical to those used in Figure 10.

Here, the PAN/magnetite nanofiber mat electrospun on a non-conductive substrate shows a clearly isotropic angle distribution (Figure 11b). PAN/magnetite on the not grounded substrate (Figure 11d) shows a clear anisotropy with a favored orientation around 90° with respect to the copper tape, i.e., perpendicular to its borders. This behavior is even stronger for the PAN/nanofiber mat spun on the grounded copper tape (Figure 11c). It must be mentioned, however, that the pure PAN nanofiber mat, electrospun on the copper tape, shows no fully isotropic angle distribution, but may indicate some favored orientations. This finding—which is not the main topic of this paper—will be investigated in the future.

These first results indicate that in general it is possible to obtain highly oriented magnetic nanofibers by electrospinning on partly conductive areas. Nevertheless, more research is necessary to optimize this process, e.g., by tailoring the polymer:nanoparticle ratio, the spinning parameters—especially the spinning duration, and the geometry of the conductive paths. It can be imagined that a fine network of thin parallel conductive lines may allow for creating fully aligned areas of magnetic nanofibers; an idea which has to be examined in the future.

## 4. Conclusions and Outlook

In this paper we report on the influence of a partly conductive substrate on wire-based electrospun magnetic nanofiber mats. Nanofiber diameters were increased by approximately a factor of 2, as compared to pure PAN nanofibers, by the addition of magnetite nanoparticles. We find that independent of the orientation of the conductive paths with respect to the electrospinning wires and a possible grounding of the copper tape, the first magnetic nanofibers are always oriented perpendicular to the borders of the conductive areas. FFT calculations and calculations of fiber angle distributions underline the strong fiber orientation along the borders of conductive areas. Elongated electrospinning, however, results in strong accumulation of the nanofibers in these regions which apparently disturb the magnetic fields responsible for the fiber orientation.

In the next experiments, this orientation process will be optimized to gain more highly oriented nanofibers by modifying the solution and spinning parameters as well as the geometry of the conductive paths. In addition, measurements of the magnetic properties will be carried out to investigate the magnetic anisotropy in addition to the optically recognizable fiber alignment.
